# Risk Factors for Post‐Endoscopic Retrograde Cholangiopancreatography Pancreatitis in Patients With Post‐Endoscopic Sphincterotomy Papillae

**DOI:** 10.1002/deo2.70322

**Published:** 2026-04-07

**Authors:** Shuhei Shibata, Katsunobu Tawada, Hideyuki Takashiro, Shinichi Tazawa, Masatoshi Usui, Hiromasa Nomoto, Hirofumi Saito

**Affiliations:** ^1^ Department of Gastroenterology Chiba Kaihin Municipal Hospital Chiba Japan; ^2^ Department of Gastroenterology Chiba University Hospital Chiba Japan

**Keywords:** body mass index, pancreatitis, retrospective studies, risk factors, sphincterotomy

## Abstract

**Objectives:**

Post‐endoscopic retrograde cholangiopancreatography (ERCP) pancreatitis (PEP) is a well‐known complication. While most reported risk factors relate to treatment‐naïve papillae, those in patients with post‐endoscopic sphincterotomy (post‐EST) papillae remain unclear. We aimed to identify risk factors for PEP in patients with a history of EST undergoing ERCP for biliary disease.

**Methods:**

We retrospectively analyzed 678 patients with prior EST who underwent ERCP between January 2017 and October 2023. PEP was diagnosed according to established criteria. Logistic regression analyses were performed to identify risk factors. To account for repeated ERCP sessions, additional analyses using cluster‐robust standard errors were conducted. Body mass index (BMI) was evaluated as both a continuous variable and according to World Health Organization categories. Prophylactic rectal diclofenac or intravenous nafamostat mesilate was administered at the operator's discretion.

**Results:**

The mean age was 75.4 years, and PEP occurred in 4.4%. Multivariate analysis identified female sex (*p* = 0.016; odds ratio [OR] 2.97), low BMI (*p* = 0.021; OR 2.94), and prolonged procedure time (*p* = 0.0048; OR 4.29) as independent risk factors. Operator experience and prophylactic agents were not significantly associated with PEP. In analyses accounting for within‐patient correlation, BMI <18.5 kg/m^2^ remained significantly associated with PEP (OR 2.73; *p* = 0.012), whereas associations with sex and procedure duration were attenuated.

**Conclusions:**

Low BMI is a consistent risk factor for PEP in patients with post‐EST papillae. The effectiveness of pharmacologic prophylaxis may differ from that in treatment‐naïve papillae and warrants prospective evaluation.

**Trial Registration:**

N/A.

## 1 Introduction

Pancreatitis is one of the most fatal complications of endoscopic retrograde cholangiopancreatography (ERCP). Post‐ERCP pancreatitis (PEP) has several risk factors. Patient‐related risk factors include suspected sphincter of Oddi dysfunction (SOD) [1, 2], female sex [1, 2], and history of PEP [2], while procedure‐related risk factors include pre‐cut sphincterotomy and multiple pancreatography [1].

Numerous reports [1, 2, 3, 4, 5, 6, 7] on the risk factors for PEP have been documented in clinical guidelines [8]. However, nearly all of these reports pertain to treatment‐naïve papillae. Endoscopic sphincterotomy (EST) is the standard treatment for biliary diseases, including choledocholithiasis, benign bile duct strictures, and malignant bile duct strictures. Although patients commonly undergo repeat ERCP for post‐EST papillae, reports on the risk factors for PEP in post‐EST papillae are lacking. Although the administration of diclofenac suppositories [9, 10, 11] and nafamostat mesilate [12, 13] before and after ERCP has been proposed to prevent PEP, studies focusing exclusively on their use in patients with post‐EST papillae are lacking. Thus, in this study, we aimed to evaluate the risk factors for PEP in patients with a history of EST who underwent ERCP for biliary disease.

## 2 Methods

### 2.1 Study Population

Between January 2017 and October 2023, 1659 patients underwent ERCP at our hospital. For analysis, we included patients with a history of EST who underwent ERCP for biliary diseases. Patients who underwent additional EST, those who underwent ERCP for gallstone pancreatitis, and those with a history of chronic pancreatitis were excluded from the study. This retrospective study was conducted in accordance with the ethical principles of the Declaration of Helsinki and was approved by the Ethics Committee of Chiba Kaihin Municipal Hospital. The ethics committee waived the requirement for informed consent owing to the retrospective design of the study.

### 2.2 ERCP Procedure

All 678 patients included in the study underwent endoscopic lithotripsy or biliary stenting; none underwent pancreatic stenting. Diclofenac suppositories or intravenous nafamostat mesilate were administered before ERCP to prevent PEP at the discretion of the attending physician. In all cases, administration was initiated at the time of departure from the ward prior to ERCP. The dose of diclofenac suppository was 50 mg, and that of intravenous nafamostat mesilate was 20 mg. Operators were classified as experts if they were board‐certified by the Japan Gastroenterological Endoscopy Society; all other operators were defined as non‐experts. All ERCP procedures performed by non‐experts were conducted under the direct supervision of experts.

### 2.3 Outcomes

The incision size of the prior EST was retrospectively categorized into three groups—small, medium, and large—based on anatomical landmarks [14]. An incision not exceeding the transverse fold was classified as small; an incision extending to the superior margin of the papillary bulge was defined as large; and an incision between these two landmarks was classified as medium.

Both the diagnosis and severity grading of PEP were determined in accordance with the 2023 Post‐ERCP Pancreatitis Guidelines [8] and 2021 Acute Pancreatitis Guidelines [15]. The diagnosis required meeting at least two of the following three criteria: (1) onset of acute upper abdominal pain and tenderness, (2) elevated levels of serum or urinary amylase, and (3) abnormal findings in the pancreas associated with acute pancreatitis, as observed on ultrasonography (US), computed tomography (CT), or magnetic resonance imaging (MRI).

### 2.4 Statistical Analysis

To identify the risk factors of PEP, univariate and multivariate analyses were conducted by incorporating clinical variables, including patient characteristics, endoscopic devices, medications, procedure duration, and operator experience. The statistical methods employed included the chi‐square test, Mann‐Whitney U test, and logistic regression analysis, as appropriate. Variables showing potential significance in univariate analysis (*p* < 0.05) were selected for multivariate analysis, which was performed via logistic regression. Because some patients underwent multiple ERCP sessions during the study period, we performed an additional analysis using logistic regression with cluster‐robust standard errors, clustering on patient ID to account for within‐patient correlation. Body mass index (BMI) was analyzed both as a continuous variable and, in sensitivity analyses, as a categorical variable based on the World Health Organization‐defined BMI categories (< 18.5, 18.5–24.9, and ≥ 25 kg/m^2^). Statistical significance was set at *p* < 0.05. All statistical analyses were performed using EZR in R Commander version 1.61, programmed by Kanda [16].

## 3 Results

Table 1 presents the baseline patient characteristics. A total of 678 patients were included in the study, with a mean age of 75.4 years, and 79.8% had undergone prior small EST. PEP occurred in 4.4% of the patients.

**TABLE 1:** Baseline characteristics of the patients.

**Age (years)**	75.4 ± 12.0
**Sex**	
Male	395 (58.2%)
Female	283 (41.8%)
**BMI** (kg/m^2^)	22.4 ± 3.79
**Medications for the prevention of PEP**	
Diclofenac suppositories	322 (47.5%)
Nafamostat mesilate	463 (68.3%)
**Incision length of previous EST**	
Small	541 (79.8%)
Medium	134 (19.8%)
Large	3 (0.4%)
**Procedural details of ERCP**	
Endoscopic biliary drainage	491 (72.4%)
Endoscopic balloon stone extraction	61 (9.6%)
Endoscopic basket stone extraction	182 (64.8%)
**ERCP procedure time (min)**	34.1 ± 25.8
**Operator** ^a^	
Expert	319 (47.1%)
Non‐Expert	359 (52.9%)
**History of PEP**	39(5.8%)
**Occurrence of PEP**	30 (4.4%)
Severe PEP	0(0%)

Abbreviations: BMI, body mass index; ERCP, endoscopic retrograde cholangiopancreatography; EST, endoscopic sphincterotomy; PEP, post‐ERCP pancreatitis.

Data are expressed as the mean ± standard deviation (SD).

^a^ Operators were classified as “Experts” if they were board‐certified by the Japan Gastroenterological Endoscopy Society; all other operators were defined as “Non‐Experts”.

Table 2 shows the results of the univariate analysis with PEP as the endpoint. This analysis identified four significant risk factors for PEP: female sex (*p* = 0.0082), low BMI (*p* = 0.022), prolonged procedure duration (*p* < 0.001), and procedures performed by non‐experts (*p* = 0.013). PEP and other clinical factors, including the administration of diclofenac suppository or nafamostat mesilate, were not significantly correlated.

**TABLE 2:** Univariate analysis of risk factors for post‐endoscopic retrograde cholangiopancreatography pancreatitis (PEP) with post‐endoscopic sphincterotomy (post‐EST) papilla.

	PEP (‐)	PEP (+)	*p‐*Value
**Sex**			
Male/Female	385/263	10/20	0.0082^a^
**Age (years)**	75.6 ± 11.7	73.6 ± 15.8	0.49^b^
**BMI (kg/m^2^)**	22.5 ± 3.78	20.1 ± 3.56	0.022^b^
**Reason for performing ERCP**			
Choledocholithiasis/Malignant biliary stricture/Benign biliary stricture	421/178/49	21/7/2	0.85^a^
**Incision length of previous EST**			
Minor/Moderate/Major	506/138/4	24/6/0	0.90^a^
**EPBD**			
Used/Not used	26/622	2/28	0.81^a^
**Total bilirubin level prior to ERCP**			
Within normal range/Elevated	360/288	19/11	0.52^a^
**History of PEP**			
Present/Absent	36/612	3/27	0.54^a^
**Biliary stents 10Fr or larger**			
Used/Not Used	11/637	1/29	1.00^a^
**Diclofenac suppositories**			
Used/Not used	306/342	16/14	0.64^a^
**Nafamostat mesilate**			
Used/Not used	442/206	21/9	1.00^a^
**Biliary drainage device**			
Used/Not used	469/179	22/8	1.00^a^
**Stone extraction balloon**			
Used/Not used	57/591	4/26	0.60^a^
**ERCP procedure time (min)**	33.3 ± 25.2	50.9 ± 32.4	<0.001^b^
**Operator**			
Expert/Non‐expert	312/336	7/23	0.013^a^

Abbreviations: BMI, body mass index; EPBD, endoscopic papillary balloon dilatation; ERCP, endoscopic retrograde cholangiopancreatography; EST, endoscopic sphincterotomy; PEP, post‐ERCP pancreatitis.

Data are expressed as the mean ± standard deviation (SD).

^a^ Chi‐square test.

^b^ Mann‐Whitney U test.

Table 3 shows the results of the multivariate analysis of the four risk factors identified in the univariate analysis. In this model, BMI and procedure duration were analyzed as continuous variables. Multivariate analysis indicated female sex (*p* = 0.016; odds ratio [OR] 2.97), low BMI (*p* = 0.021; OR 2.94), and prolonged procedure duration (*p* = 0.0048; OR 4.29) as independent risk factors for PEP. Although procedures performed by non‐experts tended to be associated with a higher incidence of PEP (*p* = 0.085, OR 2.23), this was not statistically significant.

**TABLE 3:** Multivariate analysis of risk factors for post‐endoscopic retrograde cholangiopancreatography pancreatitis (PEP) with post‐endoscopic sphincterotomy (post‐EST) papilla.

	*p*‐Value	Odds ratio^a^
**ERCP procedure time (min)**	0.0048^b^	4.29 (1.56–11.8)
**BMI (kg/m^2^)**	0.021^b^	2.94 (1.17–7.37)
**Operator (Expert)**	0.085^b^	2.23 (0.89–5.58)
**Sex (Female)**	0.016^b^	2.97 (1.32–7.66)

Abbreviations: BMI, body mass index; ERCP, endoscopic retrograde cholangiopancreatography.

Values in parentheses are percentages.

^a^ 95% confidence intervals.

^b^ Logistic regression analysis.

In an additional analysis accounting for within‐patient correlation using cluster‐robust standard errors, patients with a BMI < 18.5 kg/m^2^ had a significantly increased risk of PEP compared with those with a BMI of 18.5–24.9 kg/m^2^ (*p* = 0.012; OR 2.73). The other factors, including sex, procedure duration, and operator experience, were not significantly associated with PEP in this adjusted model (Table S1).

## 4 Discussion

Almost all previous studies investigating the risk factors for PEP [1, 2, 3, 4, 5, 6, 7] have focused on naïve papillae. PEP is thought to occur due to increased intraductal pancreatic pressure caused by factors such as SOD dysfunction, papillary edema, and pancreatic duct injection during the procedure [15]. Therefore, patients with post‐EST papillae are expected to have a lower risk of PEP. To the best of our knowledge, our study is the first to examine the risk factors for PEP in patients with previously treated papillae.

In this study, the identification of female sex as a risk factor and the lack of association between operator experience and PEP are consistent with previous reports on treatment‐naïve papillae. In such cases, female sex has been recognized as a significant risk factor for PEP [1, 2]. Although earlier studies have reported that limited ERCP experience is associated with an increased risk of PEP [17, 18], others have found no significant correlation between endoscopist experience and PEP incidence [3, 19] or between procedural volume and PEP rates across centers [19, 20]. In line with these observations, current guidelines state minimal influence of the operator's experience on the incidence of PEP when ERCP is performed under the supervision of an adequately experienced endoscopist [8]. This aligns with the findings of our multivariate analysis, which showed no significant association between endoscopist experience and PEP development. However, when within‐patient correlation due to repeated ERCP sessions was taken into account using cluster‐robust standard errors, the associations of female sex and procedure duration with PEP were attenuated. This finding underscores the importance of considering repeated procedures in this patient population.

A high BMI has been reported as a risk factor for both the occurrence and severity of pancreatitis [21, 22, 23, 24, 25, 26, 27]. In non‐gallstone acute pancreatitis, both high and low BMI are risk factors [28]. However, the mechanism by which a low BMI may contribute to PEP remains unclear, meriting further investigations. Importantly, in sensitivity analyses accounting for within‐patient correlation, low BMI remained robustly associated with an increased risk of PEP. Patients with a BMI < 18.5 kg/m^2^ had nearly a threefold higher risk of developing PEP compared with those with a BMI of 18.5–24.9 kg/m^2^. This suggests that low BMI may represent a patient‐specific vulnerability, potentially reflecting reduced pancreatic reserve or increased susceptibility to procedure‐related pancreatic injury.

Regarding prolonged procedure time as a risk factor for PEP, notably, in patients with a post‐EST papilla, SOD spasms, and the presence of difficult biliary cannulation are uncommon. Instead, repeated device manipulation during prolonged procedures may cause papillary edema, potentially leading to PEP [29].

The activation of proteolytic enzymes is thought to play a key role in PEP pathogenesis. Nafamostat mesilate, with a long half‐life, is considered effective for PEP prevention [13, 30, 31, 32]. Additionally, cyclooxygenase activation has been implicated in PEP development [33]. Consequently, rectal administration of diclofenac sodium suppository, a cyclooxygenase inhibitor, before or immediately after ERCP has been proposed as a preventive measure [9, 34, 35]. Although both diclofenac and nafamostat are effective in preventing PEP, these previous studies have largely focused on treatment‐naïve papillae, where the incidence of PEP ranges from 7.7% to 10% [35]. In contrast, the present study, which targeted patients with post‐EST papillae, revealed a relatively low PEP incidence of 4.4%. In our cohort, we did not observe a statistically significant association between the administration of diclofenac or nafamostat and the incidence of PEP. However, because the decision to administer these prophylactic agents was left to the discretion of the attending physician, the possibility of indication bias cannot be excluded. Therefore, our findings should be interpreted with caution, and they do not necessarily indicate that prophylactic administration of these agents is unnecessary in this population. Rather, our results suggest that the magnitude of benefit may differ in patients with post‐EST papillae compared with those with treatment‐naïve papillae, and further prospective studies are warranted.

This study had several limitations that warrant further consideration. First, this retrospective study had a relatively small sample size and was conducted at a single institution. Multicenter studies involving larger and more diverse populations are required to validate our findings. In addition, given the retrospective design and potential indication bias in the administration of prophylactic agents, prospective studies with standardized criteria for preventive treatment are needed to more definitively evaluate their efficacy in patients with post‐EST papillae. Furthermore, we could not evaluate the number of unintentional pancreatography and insertions into the pancreatic duct because of the retrospective design. Non‐expert endoscopists more often prefer unintentional cannulation of the pancreatic duct. However, our study has suggested that the risk of PEP is not necessarily increased even by non‐expert endoscopists. The lack of data on the frequency of inadvertent pancreatic duct cannulation and pancreatography is unlikely to have significantly affected our findings.

In conclusion, we identified female sex, low BMI, and prolonged procedure duration as independent risk factors for PEP in patients with post‐EST papillae. After accounting for within‐patient correlation, low BMI remained significantly associated with PEP, whereas the effects of sex and procedure duration were attenuated. In patients with post‐EST papillae, administration of diclofenac sodium suppositories or nafamostat mesilate was not significantly associated with PEP.

## Author Contributions

The paper was authored by Shuhei Shibata, who designed and drafted the article. Katsunobu Tawada, Hideyuki Takashiro, Shinichi Tazawa, Masatoshi Usui, Hiromasa Nomoto, and Hirofumi Saito provided critical revision of the article for important intellectual content. Katsunobu Tawada interpreted statistical analysis. Shuhei Shibata finally approved the article for submission. The final version of the manuscript was approved by all authors.

## Funding

None.

## Ethics Statement

**Approval of the research protocol by an Institutional Reviewer Board**: This study was approved by the Ethics Committee of Chiba Kaihin Municipal Hospital (Chiba, Japan).

## Conflicts of Interest

The authors declare no conflicts of interest.

The ethics committee waived the requirement for informed consent owing to the retrospective design of the study.

## Supporting information




**TABLE S1**: Sensitivity analysis accounting for within‐patient correlation. Because some patients underwent multiple ERCP sessions during the study period, we performed an additional analysis using logistic regression with cluster‐robust standard errors, clustering on patient ID to account for within‐patient correlation.
