# Supplementary material for: Risk Factors for Post‐Endoscopic Retrograde Cholangiopancreatography Pancreatitis in Patients With Post‐Endoscopic Sphincterotomy Papillae
Source: DEN Open. 2026 Apr 7;6(1):e70322. doi: 10.1002/deo2.70322 (PMC13054951; doi:10.1002/deo2.70322)
Supplement: Supplementary file 1 — TABLE S1: Sensitivity analysis accounting for within‐patient correlation. Because some patients underwent multiple ERCP sessions during the study period, we performed an additional analysis using logistic regression with cluster‐robust standard errors, clustering on patient ID to account for within‐patient correlation. [file DEO2-6-e70322-s001.docx]

**Supplementary Table S1 Sensitivity analysis accounting for within-patient correlation**

|  | ***p value*** | **Odds Ratio†** |
| --- | --- | --- |
| **BMI < 18.5 kg/m²** | 0.012* | 2.73 (1.25-5.97) |
| **BMI ≥ 25 kg/m²** | 0.52* | 0.63 (0.16-2.46) |
| **ERCP procedure time (min)** | 0.29* | 0.99 (0.98-1.01) |
| **Operator (Expert)** | 0.20* | 0.44 (0.12-1.54) |
| **Sex (Female)** | 0.61* | 0.73 (0.22-2.41) |

Reference category for BMI: 18.5–24.9 kg/m²

Values in parentheses are percentages and †95% confidence intervals.

*Logistic regression with cluster-robust standard errors (clustered by patient ID)

Abbreviations: ERCP, endoscopic retrograde cholangiopancreatography; BMI, body mass index
